# Are pelvic adhesions associated with pain, physical, emotional and functional characteristics of women presenting with chronic pelvic pain? A cluster analysis

**DOI:** 10.1186/s12905-017-0509-5

**Published:** 2018-01-08

**Authors:** Ying Cheong, Mili Saran, James William Hounslow, Isabel Claire Reading

**Affiliations:** 1 Complete Fertility Centre Southampton, University Hospitals Southampton NSH Trust, Princess Anne Hospital , Mailpoint 105, Coxford Road, Southampton, SO16 5YA UK; 2 University of Southampton Faculty of Medicine, Human Development and Health, Princess Anne Hospital, Mailpoint 815, Coxford Road, Southampton, SO16 5YA UK; 3 Primary Care and Population Sciences, Human Development and Health, University of Southampton Faculty of Medicine, Southampton General Hospital, Tremona Road, Southampton, SO16 6YD UK

**Keywords:** Adhesions, Chronic pelvic pain, Quality of life, Cluster analysis, Laparoscopy

## Abstract

**Background:**

Chronic pelvic pain is a debilitating condition. It is unknown if there is a clinical phenotype for adhesive disorders. This study aimed to determine if the presence or absence, nature, severity and extent of adhesions correlated with demographic and patient reported clinical characteristics of women presenting with CPP.

**Methods:**

Women undergoing a laparoscopy for the investigation of chronic pelvic pain were recruited prospectively; their pain and phenotypic characteristics were entered into a hierarchical cluster analysis. The groups with differing baseline clinical and operative characteristics in terms of adhesions involvement were analyzed.

**Results:**

Sixty two women were recruited where 37 had adhesions. A low correlation was found between women’s reported current pain scores and that of most severe (*r* = 0.34) or average pain experienced (*r* = 0.44) in the last 6 months. Three main groups of women with CPP were identified: Cluster 1 (*n* = 35) had moderate severity of pain, with poor average and present pain intensity; Cluster 2 (*n* = 14) had a long duration of symptoms/diagnosis, the worst current pain and worst physical, emotional and social functions; Cluster 3 (*n* = 11) had the shortest duration of pain and showed the best evidence of coping with low (good) physical, social and emotional scores. This cluster also had the highest proportion of women with adhesions (82%) compared to 51% in Cluster 1 and 71% in Cluster 2.

**Conclusions:**

In this study, we found that there is little or no correlation between patient-reported pain, physical, emotional and functional characteristics scores with the presence or absence of intra-abdominal/pelvic adhesions found during investigative laparoscopy. Most women who had adhesions had the lowest reported current pain scores.

## Background

Chronic pelvic pain (CPP) is a debilitating condition with a heterogeneous etiology, affecting in primary care, 38 per 1000 women, which is comparable with that of back pain (41 per 1000) and asthma (37 per 1000) [1]. 20% of all gynecological outpatient appointments are attributable to patients with CPP. The diagnosis, investigations and treatment of women with chronic pelvic pain are often orientated at treating organic pathology. Whilst treatment of conditions such as endometriosis may improve pain symptoms [2], women with conditions such as pelvic adhesions or apparently normal pelvises at laparoscopy (up to 55%) [3] often have a less defined treatment pathway, resulting in many of them having unexplained symptoms and being frequent attenders of clinics and hospitals [4]. Except for short-term use of progestogens or GnRHa, there are currently a limited number of effective interventions for non-endometriosis related CPP [5]. A multidisciplinary integrated, compared to traditional medical and/or surgical management has been proposed as a more effective model of care [6].

The management of pelvic adhesions in women with chronic pelvic pain is controversial; whilst some gynecologists will routinely perform adhesiolysis in the presence of adhesions, others do not. We and others have previously shown that adhesiolysis may only benefit a subgroup of yet uncharacterized patients [7, 8], although Cheong et al., 2014 was stopped before recruitment reached a statistically powered sample size due to low enrollment. Swank et al., 2003 did not report this benefit [9]. There is some evidence to suggest that dense and vascular adhesions are more likely to result in pain, and the traditional belief that adhesions attached to pain sensitive structures such as ovaries are more likely to result in more pain [7]. However, a study by Rapkin et al., 1986, did not find an association between density or site of adhesions and pelvic pain [10]. A retrospective study by Steege and Stout (1991) found the presence of psychosocial compromise was associated with a lack of salutary response with adhesiolysis [11], suggesting perhaps the ‘adhesion phenotype’ may relate, not just to the physical-mechanistic aspect of adhesions, but also to the psychosocial characteristic of the patients.

The presence, site and nature of adhesions observed intra-operatively is often unpredictable pre-operatively and whilst one expects patients with previous laparotomy to have more abdominal or pelvic adhesions than those who had previous laparoscopic surgery, laparotomy and laparoscopy are associated with comparable risks of adhesion related operative and non-operative morbidity [12, 13]. There is only moderate correlation between skin scar characteristics and intra-abdominal adhesions [14]. Moreover, significant number of patients in the aforementioned study did not have CPP and many with CPP may not have had previous surgery.

Whilst clinical phenotyping has been shown to be helpful for the management of patients with chronic pain syndromes [15–17], a clinical phenotype for adhesive disorders in terms of pain, physical, emotional and functional characteristics of women presenting with adhesions and non-adhesions related chronic pelvic pain has not yet been defined. Identifiable clinical characteristics may help facilitate better surgical decisions making and serve as better tools for pre-operative counseling.

We aim to determine if adhesions correlated with demographic and patient reported clinical characteristics of women presenting with CPP.

## Methods

This study was conducted between December 2010 and December 2014 with a total recruitment time of approximately 18 months. Participants who required a laparoscopy to investigate their chronic pelvic pain were screened and recruited. The inclusion criteria were: 1) woman’s age over 18; 2) presence of chronic pelvic pain defined as pelvic pain which is constant/cyclical in nature for greater or equal to 6 months duration; 3) written consent. The exclusion were: 1) malignancy; 2) diagnosed psychiatric disorders such as bipolar disorders, bipolar disorders etc. for which the patient has received a psychiatric diagnosis and was on medication; 3) pathology which requires urgent treatment, such as ovarian cyst or pelvic abscess; 4) women taking central nervous system stimulants; 5) hormonal treatment; 6) pregnancy and 7) known diagnosis of endometriosis.

At recruitment, pain scores were measured with the VAS (visual analogue score) from the McGill pain questionnaire. The McGill pain questionnaire is a self-report questionnaire for intensity and quality of pain [18]. Quality of life measures were obtained using SF-12 (medical outcomes study with 12 item short-form health survey), which is a short generic measure of subjective health status including12 items encompassing the self-assessment of health, physical functioning, physical role limitation, mental role limitation, social functioning, mental health and pain) and modified EHP-30 (endometriosis health profile) questionnaire for pelvic pain [19, 20]. The EHP-30 consists of core instruments on five scale scores, namely pain, control and awareness, social support, emotional well-being and self-image; it is a health related quality of life patient self-report, used to measure the wide range of effects of endometriosis.

Laparoscopic surgeons skilled in advanced laparoscopy performed the surgery where the entry into the abdomen was either via the open technique or the traditional Veress needle. CO_2_ pneumo-peritoneum was created with 20 mmHg, before the intra-umbilical insertion of the 10 mm trocar, with ot without the insertion of two or three more extra ports. The principles of microsurgery were followed during the surgery, with meticulous hemostatic control and constant irrigation to reduce the risk of tissue desiccation.

A standard proforma was used to document the patient’s history, clinical examination and operative findings with the extent, severity and site of any adhesions scored with a well-validated adhesion scoring system^15^. The information gathered regarding pain and quality of life assessment through the VAS, SF-12 and EHP-30 scores were entered into a computerized database.

The primary outcome measure was pain score (VAS) and the secondary outcomes were a) SF12 Health related quality of life scores, the modified pelvic pain EHP-30 core questionnaire and the relevant clinical outcomes which included complications and adverse events.

### Statistical analysis

Women’s pain, EHP and SF36 profile were included in a hierarchical cluster analysis. Hierarchical cluster analysis is a statistical technique which identifies those women who are similar to each other in terms of their overall profile and thus identifies data-driven rather than a-priori groups, or ‘clusters’, of women with similar attributes. It is a powerful technique to identify patterns within data which makes no statistical assumptions of normality and can be used on small sample sizes.

We then explored whether the groups identified by this method had differing baseline clinical and operative characteristics in terms of adhesions involvement. These were presented in median scores ± interquartile range.

## Results

Sixty-two women were recruited and entered into the analysis. The following were thier baseline characteristics: median age (years) and interquartile range (IQR) of 31, IQR 26–35; the median length of time since the diagnosis of chronic pelvic pain to the women entering the study was 3.7 years, IQR (0.1–9.9) and a range of 1–34 years. Fifteen women had no children and 36 of them had one or more children, and 11 women did not answer this question. Sixteen women were single, twenty-seven were co-habitating, seventeen were married and two were divorced. 37 women had adhesions involving the respective distribution (abdominal wall, 18; Adnexa, 20; uterus, 7; ovary, 12; bowel, 10) and 25 women had no adhesions. Three women who had adhesions were graded as severe and extensive, one in each cluster.

The correlation between women’s reported current pain scores and that of most severe (*r* = 0.34) or average pain experienced (*r* = 0.44) in the last 6 months was low.

**Fig. 1:**
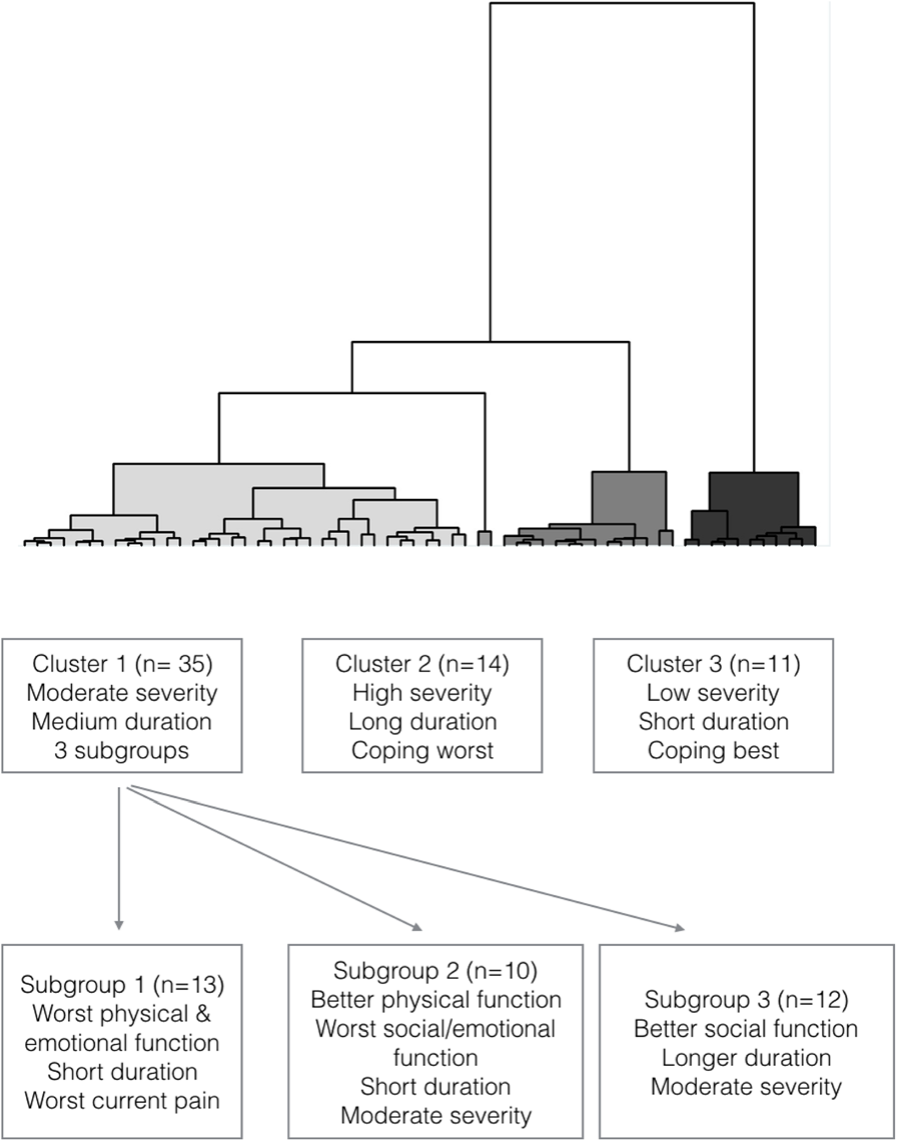
Dendogram of hierarchical cluster analysis and cluster overview and characteristics grouping

Cluster analysis of the database was performed with 62 patients’ data. Three main groups of women with CPP were identified (Cluster 1–3) (Fig. 1, Dendogram of hierarchical cluster analysis) with two women with a short duration of much milder symptoms found as outliers in a fourth cluster (*n* = 2, excluded from further analysis). Table 1 shows the cluster characteristics with the mean scores for SF36, EHP and McGill questionnaires.

**Table 1:** Hierarchical clustering of patients in accordance to their pain, modified EHP and SF 36 profile, and Cluster 1 sub-groups

Characteristics	Cluster 1 (*n* = 35)	Cluster 2 (*n* = 14)	Cluster 3 (*n* = 11)
Physical SF 36	37.5 (18.8–50)	18.75 (12.5–37.6)	87.5 (81.3–87.8)
Emotional SF 36	36.25 (28.8–43.8)	18.75 (10–25)	77.5 (68.7–82.5)
EHP Pain	56.8 (50–64)	72.7 (70–77)	29.5 (9–36)
EHP Control	75.0 (62.5–87.5)	93.75 (83.3–100)	41.7 (20.8–54.2)
EHP Emotion	58.3 (41.7–66.7)	87.5 (75–100)	8.3 (0–37.5)
EHP Social	56.3 (37.5–68.8)	84.3 (75–100)	25 (6.3–50)
EHP Image	50 (33.3–66.7)	83.3 (75–83.3)	8.3 (0–33.3)
Length of surgery/diagnosis (years)	4 (0.7–9.5)	9.9 (2.8–14.7)	2 (1.0–4.3)
Worst pain intensity in the last 6 months (0–10)	10 (9–10)	10 (9–10)	8 (6–9)
Average pain intensity in the last 6 months (0–10)	7 (6–8)	8 (7–9)	5 (4–7)
Pain score today (0–100)	34 (18–62)	67 (51–78)	16 (2–23)
Cluster 1 subgroups			
Characteristics	Subgroup 1 *n* = 13	Subgroup 2 *n* = 10	Subgroup 3 *n* = 12
Physical SF 36	18.75 (18.75–32.25)	59.4 (44–69)	37.5 (28.1–43.8)
Emotional SF 36	35 (26.3–38.75)	38.8 (32.5–61.3)	36.25 (29.4–43.8)
EHP Pain	61.3 (56.8–70.4)	52.3 (50–66)	56.8 (43.2–61.4)
EHP Control	79.2 (78–87.5)	72.9 (58.3–87.5)	62.5 (56.3–79.2)
EHP Emotion	62.5 (50–66.6)	66.7 (54.2–83.3)	43.8 (37.5–60.4)
EHP Social	50 (43.8–62.5)	71.9 (56–81)	37.5 (31.3–59.4)
EHP Image	50 (41.7–66.7)	41.7 (33.3–66.7)	41.7 (4.2–66.6)
Length of surgery/diagnosis (years)	1.6 (0.5–4.4)	1.8 (0.7–3.8)	9.6 (6.3–14.6)
Worst pain intensity in the last 6 months (0–10)	10 (9–10)	10 (9–10)	10 (8.5–10)
Average pain intensity in the last 6 months (0–10)	8 (7–8)	6.5 (6–8)	7 (6.5–8)

Patients who fell into Cluster 1 (*n* = 35) to the left of the dendrogram in Fig. 1 had moderate severity of pain, with their worst pain intensity in the last 6 months (VAS score, median ± IQR) at 10 (9, 10), their average pain intensity in the last 6 months at 7 (6–8), and their present pain at 34 (18–62). Cluster 2 (*n* = 14) in the middle of the dendrogram consisted of women who had chronic pelvic pain symptoms diagnosed for the longest duration (comparative to the cohort), at 9.9 (2.8–14.7) years, the worst current pain (67 (51–78)) VAS scores and worst physical, emotional and social functions. In Cluster 3, to the right of the dendrogram, women had the shortest duration of pain history (2 years, (1–4.3)) and showed the best evidence of coping with low (good) physical, social and emotional scores.

Cluster 1 was the largest cluster identified, although Fig. 1 illustrates that this group was made up of three distinct smaller clusters of similar size. These three subgroups comprised those with a short duration of symptoms but worst pain, and physical function and poor social function (Subgroup 1, *n* = 13); those with a short duration of pain and better physical function but worse social and emotional function (Subgroup 2, *n* = 10); and those with a moderate pain severity and a longer duration of symptoms but better social function (Subgroup 3, *n* = 12).

The site, presence or absence of adhesions is shown in Table 2. There was no correlation of the nature of pain and the site or type of adhesions present during laparoscopy. 51% (*n* = 18), 71% (n = 10) and 82% (*n* = 9) of women in Clusters 1 to 3 respectively had adhesions. Thus, the cluster with the lowest current median pain scores (Cluster 3) had the highest proportion of women with adhesions.

**Table 2:** Distribution of adhesions in each cluster

Sites	Cluster 1 (*n* = 35)	Cluster 2 (*n* = 14)	Cluster 3 (*n* = 11)
Abdominal wall	11	5	3
Ovary	7	1	2
Bowel	6	2	3
Adnexa	8	2	6
Uterus	6	0	1
Total number of women with adhesions in each group	18 (51%)	10 (71%)	9 (82%)

## Discussion

In this study, we found that there is little or no correlation with the presence or absence of intra-abdominal/pelvic adhesions during diagnostic laparoscopy and the patient reported pain, physical, emotional and functional characteristics. The greatest proportion of women who had the least reported current pain. The longest duration of CPP captured in our study was 34 years. We also did not find any correlation with the nature of pain (severity, duration and intensity of pain) and the site or type of adhesions present during laparoscopy in women presenting with chronic pelvic pain. We found the correlation between the reported current pain scores and that of most severe (*r* = 0.34) or average pain experienced (*r* = 0.44) in the last 6 months captured by the questionnaires to be low which is not surprising given that the characteristics of pain generally vary over time. As endometriosis pain may be more inflammatory and neurogenic in origin, we did not include this group of women in our analysis.

Women who presented at our gynecological service fell into three clinical characteristics clusters; namely those currently with a low severity of pain, short duration and were apparently coping best, those who have moderate severity of pain, with medium duration of symptoms with varying coping scores, and the third cluster whose current symptoms are the most severe, had pain for a long duration and appeared to be coping worst. It is not known if the social, emotional and physical scores deteriorate with the duration of pain symptoms in chronic pelvic pain sufferers, that is, if these cluster characteristics are progressive for any individual, but such a hypothesis is highly plausible and will have to be further explored by longitudinal studies. However, we found some subgroups of women who, despite long durations of pain had good social and emotional scores and vice versa, suggesting that women display a variety of characteristics that do not necessarily correlate with each other in terms of severity or duration.

Our cluster analysis has highlighted that women with CPP can present with quite distinct profiles of characteristics and the traditional gynecological approach to history taking and management strategy will not be adequate for their pain and expectation management. A cognitive behavioral-based assessment, which involved the assessment of cognitive, emotional, behavioral and physical assessment of the patient has been advocated to facilitate the initial assessment and pain management referral [21], although due to various organizational and financial reasons, such a model of management has not been trialed nor is routinely used in the management of CPP.

Recall bias may account for the lack of correlation of patient reported current pain scores compared to worst or average pain in the last 6 months, and perhaps future research can explore more continuous monitoring of the CPP patient to better and more accurately reflect the physical, functional and emotional aspects of sufferers of CPP. Whilst the sample size of this study is limited to 62 participants, the use of hierarchical cluster analysis is a useful exploratory tool to identify possible patterns in the data which makes no assumptions of normality. Our study only focused on the presence and absence of adhesions, and had not specifically teased out conditions such as abdominal wall and pelvic floor myofascial pain, neuropathic pain, irritable bowel syndrome, and bladder pain syndrome, which could have increased the heterogeneity of our study population. However, a significant majority of practitioners in our practice, would not normally carry out specific tests to include or exclude these conditions and hence such details were not available to us.

## Conclusions

In women with chronic pelvic pain, clinical characteristics depicted by quality of life questionnaires SF36, EHP-30 and McGill pain scores do not help differentiate those with adhesions from those without prior to surgery. However, our cluster analysis highlights that women with CPP can present with quite distinct profiles of characteristics. It is, therefore, high time we place due attention to the fact that history and evaluation of patients with chronic pelvic pain must include cognitive, emotional behavior and other physical assessments prior to embarking on surgical management.

## Abbreviations

CPP: Chronic pelvic pain
VAS: Visual analogue score
